# Vacuum stent: a game-changer in endoscopic multistep management of leakage following bariatric surgery

**DOI:** 10.1055/a-2320-1970

**Published:** 2024-05-29

**Authors:** Marina La Marca, Maria Luisa Bianchi, Andrea Lucchi, Laura Agostinelli, Giulia Vitali, Angelo De Padova, Marco Di Marco

**Affiliations:** 1 26208 Gastroenterology and Digestive Endoscopy Unit, AUSL della Romagna Rimini, Rimini, Italy; 2 26208 Department of Surgery, Ceccarini Hospital, AUSL della Romagna, Riccione, Italy

The most common adverse events following laparoscopic sleeve gastrectomy are leaks in the staple line, which occur in up to 2% of cases 1 . Apart from surgical repair, various endoscopic treatment options have been explored such as placement of self-expandable metal stents (SEMSs) 2 . Recently, endoscopic vacuum therapy (EVT) has become a valid alternative owing to a clinical success rate of up to 95%, shorter treatment duration, and lower mortality rate 3 . However, intraluminal EVT in the upper gastrointestinal tract prevents early enteral nutrition. The VACStent (Micro-Tech Europe, Dusseldorf, Germany) is a new treatment option that overcomes the drawbacks of endoluminal EVT and combines EVT with the advantages of covered stenting.

We report the case of a 30-year-old woman with moderate obesity who underwent laparoscopic sleeve gastrectomy in a private hospital and developed a 30-mm vertical staple-line leakage within 1 week. After the insertion of an abdominal drain, a fully covered esophageal SEMS was placed and secured with two clips. However, early migration of the stent and critical patient conditions led to the adoption of VACStent treatment to obtain quick closure of leaks and improvement in the patient outcome. After consecutive treatment with three VACStents over 13 days, the leak was almost completely healed ( Video 1 , Fig. 1 ). After multidisciplinary discussion, another fully covered SEMS was placed and left for 4 weeks; the subsequent computed tomography scan plus oral contrast agent demonstrated the complete closure of leaks and resolution of the perigastric abscess ( Fig. 2 ). Radiological and endoscopic follow-up revealed anastomotic strictures causing dysphagia that were successfully treated with 2 sessions of endoscopic pneumatic dilation.

**Fig. 1:**
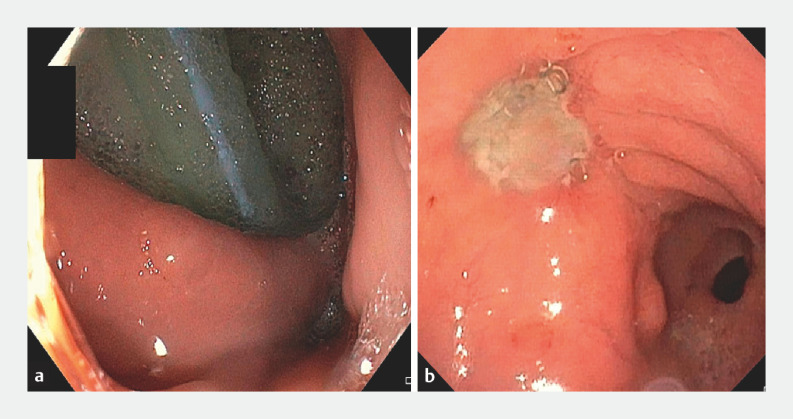
Management of leakage following bariatric surgery in a 30-year-old woman: endoscopic views. **a** Early vertical staple-line leakage about 30 mm in diameter after surgical placement of an intra-abdominal drain. **b** Residual granulation tissue over the fistula, which is completely healed after 2 months.

**Fig. 2:**
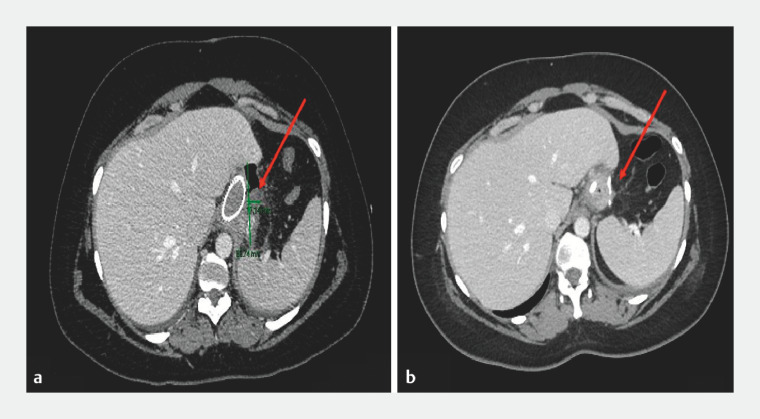
Computed tomography showing (arrowed): **a** huge perigastric abscess with visible air bubbles after placement of the first self-expanding metal stent (SEMS); **b** complete resolution of the collection after 2 months.

Endoscopic management of a complex staple line leak after laparoscopic sleeve gastrectomy, using fully covered esophageal self-expanding metal stents (SEMSs) and vacuum-stents.Video 1

Overall, managing adverse events following bariatric surgery requires a multidisciplinary approach. The combined surgical and endoscopic treatment discussed was a good option for patients with complex staple line leakage. Early use of EVT via the vacuum stent was beneficial for this patient.

Endoscopy_UCTN_Code_TTT_1AO_2AI

## Notes

Endoscopy E-Videos https://eref.thieme.de/e-videos
*E-Videos* is an open access online section of the journal *Endoscopy* , reporting on interesting cases and new techniques in gastroenterological endoscopy. All papers include a high-quality video and are published with a Creative Commons CC-BY license. Endoscopy E-Videos qualify for HINARI discounts and waivers and eligibility is automatically checked during the submission process. We grant 100% waivers to articles whose corresponding authors are based in Group A countries and 50% waivers to those who are based in Group B countries as classified by Research4Life (see: https://www.research4life.org/access/eligibility/ ). This section has its own submission website at https://mc.manuscriptcentral.com/e-videos .
